# Insights into How Plant-Derived Extracts and Compounds Can Help in the Prevention and Treatment of Keloid Disease: Established and Emerging Therapeutic Targets

**DOI:** 10.3390/ijms25021235

**Published:** 2024-01-19

**Authors:** Yong Chool Boo

**Affiliations:** 1 Department of Molecular Medicine, School of Medicine, Kyungpook National University, 680 Gukchaebosang-ro, Jung-gu, Daegu 41944, Republic of Korea; ycboo@knu.ac.kr; 2 BK21 Plus KNU Biomedical Convergence Program, Department of Biomedical Science, The Graduate School, Kyungpook National University, 680 Gukchaebosang-ro, Jung-gu, Daegu 41944, Republic of Korea; 3 Cell and Matrix Research Institute, Kyungpook National University, 680 Gukchaebosang-ro, Jung-gu, Daegu 41944, Republic of Korea

**Keywords:** keloid, transforming growth factor β, TGF-β, small mothers against decapentaplegic, SMAD, phenolic compounds, terpenoids, alkaloids, natural products, plant extracts

## Abstract

Keloid is a disease in which fibroblasts abnormally proliferate and synthesize excessive amounts of extracellular matrix, including collagen and fibronectin, during the healing process of skin wounds, causing larger scars that exceed the boundaries of the original wound. Currently, surgical excision, cryotherapy, radiation, laser treatment, photodynamic therapy, pressure therapy, silicone gel sheeting, and pharmacotherapy are used alone or in combinations to treat this disease, but the outcomes are usually unsatisfactory. The purpose of this review is to examine whether natural products can help treat keloid disease. I introduce well-established therapeutic targets for this disease and various other emerging therapeutic targets that have been proposed based on the phenotypic difference between keloid-derived fibroblasts (KFs) and normal epidermal fibroblasts (NFs). We then present recent studies on the biological effects of various plant-derived extracts and compounds on KFs and NFs. Associated ex vivo, in vivo, and clinical studies are also presented. Finally, we discuss the mechanisms of action of the plant-derived extracts and compounds, the pros and cons, and the future tasks for natural product-based therapy for keloid disease, as compared with existing other therapies. Extracts of *Astragalus membranaceus*, *Salvia miltiorrhiza*, *Aneilema keisak*, *Galla Chinensis*, *Lycium chinense*, *Physalis angulate*, *Allium sepa*, and *Camellia sinensis* appear to modulate cell proliferation, migration, and/or extracellular matrix (ECM) production in KFs, supporting their therapeutic potential. Various phenolic compounds, terpenoids, alkaloids, and other plant-derived compounds could modulate different cell signaling pathways associated with the pathogenesis of keloids. For now, many studies are limited to in vitro experiments; additional research and development are needed to proceed to clinical trials. Many emerging therapeutic targets could accelerate the discovery of plant-derived substances for the prevention and treatment of keloid disease. I hope that this review will bridge past, present, and future research on this subject and provide insight into new therapeutic targets and pharmaceuticals, aiming for effective keloid treatment.

## 1. Introduction

The skin has developed an efficient mechanism for healing wounds consisting of four main stages: hemostasis, inflammation, proliferation, and extracellular matrix (ECM) remodeling [1]. Dermal fibroblasts play diverse pivotal roles in the wound healing process [2]. The cells produce the majority of collagens and other ECM components and regulate ECM remodeling [3]. Although fibroblasts produce ECM components, their functions are conversely affected by those ECM components [4]. The interaction between fibroblasts and ECM components is considered a form of autocrine regulation of the wound healing process, as it is mainly mediated by the members of the transforming growth factor-β (TGF-β) superfamily [5,6].

Keloid is a type of scarring that occurs due to abnormally high cell proliferation and the excessive accumulation of ECM during the healing process of a skin injury [7,8]. Keloids are similar to hypertrophic scars in that they involve the proliferation of dermal fibroblasts and accumulation of ECM, but keloids differ in that they grow beyond the boundaries of the initial injury [9]. In hypertrophic scars, collagen or other ECM components show a wavy or spiral pattern arranged in a specific direction, but, in keloids, ECM does not show a consistent or regular pattern [10]. Additionally, hypertrophic scars gradually become smaller and lessen over time, but keloids are different in that they grow larger or persist [11,12]. Therefore, keloids are sometimes classified as benign fibroproliferative skin tumors [13].

Keloids and hypertrophic scars are both aesthetically disfiguring and functionally defective and can cause pruritus (itchiness), pressure, or pain depending on their shape, size, and location [11,14]. In treating keloids, surgical excision, cryotherapy, radiation, laser treatment, photodynamic therapy, pressure therapy, silicone gel sheeting, and pharmacotherapy are currently used alone or in combinations [15,16,17,18,19]. In pharmacotherapy, steroids, retinoids, interferons, imiquimod, etc. are administered by intralesional injection or topical application [15,16,17,18,19]. However, the outcomes are usually unsatisfactory, and further technical development is needed.

Studies have been conducted extensively to prevent and treat keloids by using various natural products in parallel with the application of various surgical, physical, and pharmacological therapies [15,18]. Single or mixed plant extracts have been traditionally used in treating keloid scars, and recent studies have shown that various plant-derived compounds have biological activities potentially applicable to therapeutics for keloid disease [20].

The purpose of this review is to examine whether natural products can help treat keloid scars. In this review, we first introduce well-established and newly proposed therapeutic targets useful for the development of keloid treatment drugs. We then present recent studies on the biological activities and mechanisms of action of natural products relevant to the treatment of keloids. Finally, we discuss the limitations of the current studies and future perspectives for the development of natural-product-based keloid therapy. We hope that this review will provide insight into new therapeutic targets and pharmaceuticals aimed at effective keloid treatment.

## 2. Methods: Study Search and Selection

The primary purpose of this review is to provide comprehensive information to researchers studying natural-product-based keloid treatments. We used the Pubmed database (https://pubmed.ncbi.nlm.nih.gov/, accessed on 9 January 2024) to search for studies on plant-derived extracts and compounds related to keloids with the following key terms: keloid[TI] AND (polyphenol OR phenolic OR flavonoid OR terpenoid OR alkaloid OR natural product OR extract[TI]). Through this search process, 78 papers written in English with keloid in the title and polyphenol, phenolic, flavonoid, terpenoid, alkaloid, or natural product in the total bibliographic information, or extract in the title, were selected. Unlike a systematic review, strict inclusion and exclusion criteria were not applied in this narrative review, and the scientific level and reliability of each paper were not subjectively evaluated. Most selected papers were cited and discussed in the appropriate sections(s) according to their contents, excluding 5 old papers published between 1951 and 1983, 5 case reports, and 1 commentary.

## 3. Established and Emerging Therapeutic Targets

This section introduces the well-established therapeutic targets that have been widely used in keloid research and emerging therapeutic targets that have recently been proposed based on the phenotypic differences between keloid-derived cells (KFs) and normal skin fibroblasts (NFs). The distinction between established and emerging targets is arbitrary.

### 3.1. Cell Proliferation

Viewing keloid as a type of benign tumor accompanied by an excessive proliferation of fibroblasts, attempts are being made to prevent cell division or proliferation. However, non-selective inhibition of cell proliferation has limited application due to its high risk. Mitomycin C is an anti-neoplastic compound originating from *Streptomyces caespitosus* that suppresses cell proliferation by chemically modifying DNA. 5-Fluoro uracil is an irreversible inhibitor of thymidine synthase, which suppresses cell proliferation by inhibiting DNA synthesis. The topical application and intralesional injection of mitomycin C and 5-fluoro uracil have been applied in treating keloids with significant improvements [21,22].

### 3.2. Cell Apoptosis

Compared with hypertrophic-scar-derived fibroblasts (HTFs) and NFs, KFs are highly resistant to apoptosis induced by serum depletion and the FAS ligand (antibody) [23]. External TGF-β1, but not TGF-β2, epidermal growth factor (EGF), or platelet-derived growth factor (PDGF), enhance the resistance of HTFs and NFs to FAS receptor-mediated apoptosis. Neutralizing antibodies to TGF-β1, but not TGF-β2, abrogates the resistance of KFs to apoptosis. These findings suggest that KFs are refractory to FAS receptor-mediated apoptosis due to a TGF-β1-dependent mechanism, and that this phenotype could contribute to high cellularity in keloids. KFs also show stronger drug resistance to vincristine and higher survival compared to NFs [24]. In contrast, NFs and KFs similarly undergo apoptosis when exposed to staurosporine or *N*-benzoylstaurosporine [25].

Despite the relatively strong resistance to apoptosis of KFs, apoptosis inducers may be an option for keloid treatment, depending on how they are applied. Clinical studies and animal experimental studies have reported the effective treatment of keloid through the intralesional injection of bleomycin, a glycopeptide isolate of *Streptomyces verticillus*, or triamcinolone acetonide, a steroid drug [26,27].

### 3.3. TGF-β Signaling Pathway

In the canonical TGF-β signaling pathway mediated by TGF beta receptor (TGFβR) 1 and 2, both small mothers against decapentaplegic (SMAD) 2 and SMAD3 (called receptor-regulated SMADs or R-SMADs) are phosphorylated by the protein kinase activity of TGFβR1 and bind to SMAD4 (called common partner SMADs or co-SMADs) to form a trimeric transcription factor that induces the transcription of target genes [28]. The target genes include SMAD 7 (called inhibitory SMADs or I-SMAD), which competes for TGFβR1 and thereby blocks TGF-β signaling in a negative feedback loop [29]. The activation of TGFβR also can stimulate SMAD-independent non-canonical pathways, such as the Ras small G protein/c-Raf protein kinase/mitogen-activated protein kinase kinase (MEK)/extracellular signal-regulated kinase (ERK) pathway, the phosphoinositide 3-kinase (PI3K)/protein kinase B (PKB, also called AKT)/mammalian target of rapamycin (mTOR)/ribosomal S6 kinase (p70S6K) pathway, and other relevant pathways [30].

Activation of either SMAD-dependent or -independent pathways results in the increased transcription of target genes, such as collagen, fibronectin, and other ECM components [31,32]. Therefore, targeting these TGF-β signaling pathways will inhibit cell proliferation and collagen synthesis, which can help prevent and treat keloids.

### 3.4. Collagen Synthesis Pathway

The synthesis and secretion of collagens occur via multiple steps [33,34]. After the transcription of collagen genes in the nucleus, their mRNA is transported to the cytoplasm and translated to synthesize a pre-procollagen protein [35]. The pre-procollagen is post-translationally modified to procollagen in the endoplasmic reticulum through the deletion of the *N*-terminal signal peptide, the hydroxylation of certain proline and lysine residues, and the glycosylation of certain lysine residues [36]. The hydroxylation reactions are catalyzed by 2-oxoglutarate- and ascorbic acid-dependent dioxygenases, such as prolyl 4-hydroxylase, prolyl 3-hydroxylase, and lysyl 5-hydroxylase [37,38]. Three molecules of procollagen are combined in parallel to form a left-twisted triple helix [39]. The triple helix of procollagen is further modified in the Golgi apparatus, assembled into secretory vesicles, and secreted out of the cell [40]. Both ends of the triple helix are removed outside the cells to generate tropocollagens, which are then assembled in parallel via covalent bonds to form collagen fibrils [41]. Targeting not only the transcription stage of the collagen gene but also the translation and post-translational modification stages is a useful strategy to inhibit the synthesis of collagen fibrils.

Protocatechuic acid (also called 3,4-dihydroxybenzoic acid) potently inhibits the activity of prolyl 4-hydroxylase in vitro [42]. Ethyl-3,4-dihydroxybenzoate, a hydrophobic derivative of 3,4-dihydroxybenzoic acid, reduces prolyl 4-hydroxylase activity and 4-hydroxyproline synthesis in cells [43]. As a result, it markedly reduces the synthesis and secretion of procollagens 1 and 3 without affecting their steady-state mRNA levels, cell viability, proliferation, or the synthesis of non-collagenous proteins. Thus, it is feasible to reduce procollagen production by inhibiting post-translational proline hydroxylation with ethyl-3,4-dihydroxybenzoate or other inhibitors.

### 3.5. Nuclear Factor Kappa B (NF-κB) Signaling Pathway

The NF-κB signaling pathway is involved in various immune and inflammatory responses in cells [44,45]. Normally, the NF-κB proteins, such as NF-κB1 (p50) and RelA (p65), are sequestered by NF-κB inhibitory proteins (IκBα) in the cytoplasm, but once cells are stimulated the inhibitory proteins are degraded in the proteasome following the phosphorylation by an IκB kinase (IKK) complex activated by the signals transmitted through the TNF receptor (TNFR) superfamily members or other relevant receptors. Then, the freed NF-κB1 (p50)/RelA (p65) dimers undergo nuclear translocation to stimulate target gene expression [44].

Tumor necrosis factor-alpha (TNF-α) activates the NF-κB pathways in KFs with higher sensitivity than in NFs [46,47]. Aspirin inhibits the phosphorylation and degradation of IκBα and the nuclear translocation of NF-κB, attenuates TNF-α-induced NF-κB activation and proliferation, and increases the apoptosis of KFs [46]. The anti-inflammatory drug lysine acetylsalicylate also inhibits the proliferation of KFs and the expression of procollagen 1/3 [48]. Thus, the inhibition of NF-κB could be an attractive therapeutic approach to treating keloids [49].

### 3.6. Wnt Signaling Pathway

In the canonical Wnt pathway, the stability of β-catenin is negatively regulated by phosphorylation by glycogen synthase kinase 3 beta (GSK3β) [50]. The Wnt signal enhances the binding of frequently rearranged in advanced T-cell lymphomas 1 (FRAT1) to GSK3β in the axin complex to inhibit the GSK3β-dependent phosphorylation of β-catenin, which results in the stabilization and nuclear translocation of β-catenin and the induction of target gene expression [51]. The target genes include dickkopf-1 (DKK1), which is an antagonistic inhibitor preventing the over-activation of the Wnt pathway in a negative feedback loop [52]. TGF-β1 can downregulate DKK1 by an ERK-dependent mechanism and thereby activate the Wnt pathway, supporting cooperation between TGF-β and Wnt signaling [53].

The Wnt signaling pathway appears to play a pivotal role in the pathogenesis of hypertrophic scars and keloids [54]. The protein level of β-catenin is elevated in hypertrophic scar and keloid tissues. A Wnt inhibitor, secreted frizzled-related protein 1 (SFRP1) is downregulated in KFs [55]. Treatment with Wnt3a increases β-catenin-mediated gene transcription, fibronectin expression, and the cellular growth of NFs and KFs. The ectopic expression of SFRP1 attenuates Wnt3a-induced phenotypic changes in KFs. Thus, the Wnt signaling pathway can provide a therapeutic target for treating keloid scarring.

### 3.7. Janus Kinase (JAK)/Signal Transducer and Activator of Transcription (STAT) Signaling Pathway

The JAK/STAT pathway regulates a wide range of cellular functions, such as proliferation, differentiation, migration, apoptosis, etc., in response to various cytokines and other stimuli [56]. Recently, as the importance of the JAK-STAT pathway in the pathogenesis of keloids has been recognized, studies targeting this pathway for keloid treatments are expanding [57,58].

The phosphorylation level of STAT3 is higher in KFs compared to NFs, and the pharmacological inhibition of the JAK2/STAT3 pathway or silencing RNA (siRNA)-mediated STAT3 depletion attenuates KF proliferation, migration, and collagen production [59]. The treatment of KFs with ASC-J9, a STAT3 inhibitor, suppresses the expression of COL1A1 and fibronectin 1 by inhibiting STAT3 expression and phosphorylation [60]. RNA-Seq analysis revealed that ASC-J9 downregulates the gene expression associated with inflammation, the production of nitric oxide and reactive oxygen species (ROS), and ECM synthesis in KFs. ASC-J9 attenuates cell division, collagen fibril organization, and interleukin-6 (IL-6) expression while increasing heme oxygenase 1 (HMOX1) expression in KFs. ASC-J9 induces the formation of multilamellar bodies and the expression of autophagy-related genes in KFs, indicating the occurrence of autophagy. These findings suggest that the ROS/STAT3/IL-6 axis may represent a potential therapeutic target to suppress cell proliferation and ECM production in KFs.

### 3.8. Hypoxia-Inducible Factor (HIF)-1α

Hypoxia and HIF-1α promote keloid development through the activation of the TGF-β/SMAD and the toll-like receptor (TLR) 4/NF-κB pathways and increasing collagen production [61,62]. Hypoxia has been shown to promote proliferation and inhibit the apoptosis of KFs via the HIF-1α-dependent mechanism, and these responses could be modulated pharmacologically using resveratrol [63]. Thus, research on the role of HIF-1α in keloid pathogenesis would provide a new opportunity to develop therapeutic approaches [64].

### 3.9. Prostaglandin (PG) E_2_

Macrophage migration inhibitory factor (MAF), a pluripotent cytokine, enhances cyclooxygenase (COX) 2 activity and PGE_2_ production more highly in NFs than in KFs [65]. Consistently, the expression levels of E prostanoid receptor 2 are higher in NFs compared to KFs. Because forskolin, an adenylyl cyclase activator, decreases collagen expression levels in both NFs and KFs, it is suggested that cyclic AMP negatively regulates collagen synthesis. Higher levels of PGE_2_ and consequentially high levels of cyclic AMP (cAMP) may be associated with the lower levels of collagen expression in NFs compared to KFs. Although the extent to which DNA synthesis is inhibited by external PGE_2_ in NFs and KFs are similar [66], collagen gene expression is reduced more sensitively in NFs than in KFs [65]. Thus, the difference in collagen synthesis activity between NFs and KFs may be attributable, in part, to the difference in the activity of the PGE_2_-dependent pathway.

### 3.10. Aldo-Keto Reductase Family 1 Member B10 (AKR1B10)

A laser capture microdissection-based whole-genome microarray analysis discovered that AKR1B10 is highly upregulated in the keloid epidermis, suggesting that the altered metabolism of retinoic acid may be associated with keloid pathogenesis [67]. AKR1B10 transfection into normal epidermal keratinocytes reproduces the expression pattern of retinoic acid metabolic enzymes observed in the keloid epidermis [67]. Cotransfection of AKR1B10 with a luciferase reporter plasmid results in the reduced activity of the retinoic acid response element (RARE) in cells [67]. Secreted factors from AKR1B10-overexpressing keratinocytes upregulate TGF-β1, TGF-β2, and collagens 1 and 3 in both KFs and NFs [67]. These findings suggest that impaired retinoic acid synthesis in epidermal keratinocytes may cause the pathogenesis of keloid disease and that AKR1B10 could be a potential molecular target for the treatment of keloid disease.

### 3.11. High-Mobility Group Box 1 (HMGB1)

HMGB1 was originally identified as a non-histone DNA-binding protein that regulates chromatin structure and gene expression in the nucleus [68]. It can be secreted out of cells, where it binds to receptors for advanced glycation end-products (RAGE) or TLRs to regulate various cell functions under pathological conditions, such as fibrosis [69].

KFs show high expression levels of HMGB1 and enhanced autophagy compared to NFs [70]. External TGF-β1 and HMGB1 increase the migration and autophagy of both cells [70,71]. HMGB1 increases the expression levels of ERK1/2, AKT, and NF-κB. Glycyrrhizin (also called glycyrrhizic acid), an HMGB1 inhibitor, suppresses the cell migration, autophagy, and the RAGE-mediated MAPK and NF-κB signaling pathways stimulated by HMGB1 [70,71]. Thus, targeting HMGB1 can be a promising approach to managing keloids.

### 3.12. Sphingosine-1-Phosphate (S1P) Signaling Pathway

S1P-induced mitogen-activated protein kinase (MAPK) pathways appear to be involved in keloid formation [72]. The mRNA and protein expression levels of the sphingosine-1-phosphate receptor (S1PR) 1 and 2 are higher in keloid tissues than in normal tissues. S1P stimulates the expression of COL1A1 (collagen type 1, α-1 chain) and the phosphorylation of c-Jun N-terminal kinase (JNK)/ERK in KFs to higher levels than in NFs. The S1P-induced effects are attenuated by an inhibitor of ERK (PD98059), JNK (SP600125), S1PR1 (W146), or S1PR2 (JTE013). SIP also can stimulate other signaling pathways, including the PI3K-AKT pathway [73].

The SiRNA-mediated gene silencing of S1PR1 decreases the cell proliferation, migration, invasion, and fibrosis of KFs, which is associated with the reduced phosphorylation and activity of PI3K and AKT [74]. Since activation of the S1P-dependent signaling pathway can cause cell proliferation and collagen synthesis, targeting this signaling pathway would provide new possibilities in keloid therapeutics. Isorhamnetin has been identified as a new inhibitor directly interacting with S1PR1 in a molecular docking study [74].

### 3.13. Activins and Inhibins

Activins and inhibins, which belong to the TGF-β superfamily, are dimeric proteins with opposite biological effects on the synthesis and secretion of the follicle-stimulating hormone [75]. The activin system plays critical roles in diverse cell functions, such as proliferation, differentiation, apoptosis, and metabolism [75]. KFs produce much higher levels of activin-A compared to NFs [76]. Exogenous activin-A stimulates cell proliferation and upregulates ECM components, namely collagen, fibronectin, and alpha-smooth muscle actin (α-SMA) in NFs and KFs, whereas treatment with follistatin blocks the stimulatory effects of activin on ECM components [76].

The expression levels of inhibin beta A (INHBA), a subunit of both activin and inhibin, are significantly higher in KFs than in NFs [77]. The expression levels of INHBA in KFs are lowered by SR11302, an inhibitor of the activator protein-1 (AP-1) transcription factor. Treatment with follistatin, an activin-binding protein that neutralizes TGF-β superfamily members, downregulates activin, connective tissue growth factor (CTGF), and various matrix-related genes in KFs [77]. CTGF plays a pivotal role in the regulation of cell proliferation, differentiation, and fibrogenesis [78]. Thus, the activin system could be a useful therapeutic target in managing keloids and other fibrotic diseases.

### 3.14. Growth Differentiation Factor (GDF) 9 Signaling Pathway

GDF-9 is a member of the TGFβ superfamily that binds to TGFβR1 (also called the activin receptor-like kinase 5, ALK5) to transmit signals [79]. Comparative analysis of gene expression in the fibroblasts derived from the peripheral area and central area of keloids using a TGF-β superfamily signaling gene chip identified GDF-9 as one of the differentially expressed genes which was highly upregulated in the peripheral area responsible for keloid invasion [80].

KFs with higher proliferation rates show higher GDF-9 expression levels than NFs; the overexpression of GDF-9 promotes the proliferation of NFs, and the siRNA-mediated depletion of GDF-9 decreases the proliferation of KFs [81]. GDF-9 silencing attenuates the cell cycle, lowers the expression levels of COX-2, vascular endothelial growth factor (VEGF) C, MMP2, and MMP9, and inhibits the phosphorylation of SMAD2 and SMAD3 without affecting the expression levels of TGF-β1 in KFs [81]. Recombinant human GDF-9 (rhGDF-9) enhances the proliferation, migration, and invasion of KFs without increasing collagen expression [82]. It also induces phosphorylation of SMAD2 and SMAD3 at the linker regions, which are different from the regions phosphorylated in response to TGF-β1 [82].

These findings suggest that the roles played by TGF-β1 and GDF-9 in keloid formation are overlapping only partly, and that their signaling processes are different from each other. Therefore, GDF-9 provides a therapeutic target to control the cellularity of keloids, especially by inhibiting the proliferation of KFs.

## 4. Biological Effects of Existing Therapy on Cells

**Figure 1:**
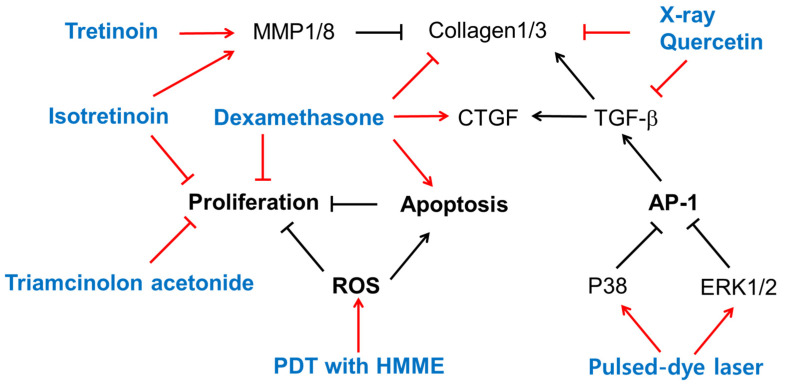
The biological effects of several existing treatments and quercetin on keloid-derived fibroblasts (KFs). Sharp arrows indicate stimulation or upregulation and blunted arrows indicate inhibition or downregulation. Black lines indicate the interaction between cellular factors and events (black letters) and red lines indicate the effects of various treatments (blue letters).

This section presents experimental findings from in vitro studies on the biological activities of currently clinically used treatments and/or quercetin at the cellular level, as summarized in Figure 1. Its purpose is to facilitate a comparison with the experimental results of the natural product research that will be introduced in the next section.

### 4.1. Retinoids

Treatment with tretinoin (all-trans-retinoic acid) or isotretinoin (13-cis-retinoic acid) reduced collagen production in KFs cultured in vitro [83]. Both retinoids did not affect the activity of prolyl hydroxylase, but markedly reduced collagenase activity and markedly enhanced the activity of an elastase-like neutral protease, indicating the retinoids’ differential modulation of collagen metabolism in KFs [83]. The mRNA and protein levels of MMP1 and MMP8 were higher in NFs, and those of MMP13 were higher in KFs [84]. Tretinoin increased the mRNA levels of MMP1 and MMP8, but not MMP13, in only NFs, and increased the mRNA levels of MMP13, but not MMP1 or MMP8, in only KFs [84]. Thus, tretinoin could reverse the altered expression profile of MMPs in KFs.

### 4.2. Steroids

Dexamethasone increased keloid apoptosis and decreased keloid proliferation and collagen synthesis, but induced CTGF overexpression [85]. Treatment with isotretinoin or triamcinolone acetonide, a glucocorticoid, significantly inhibited the growth of keloid and embryonal human skin fibroblasts in vitro, and the effect was stronger when the drugs were used in combination [86].

### 4.3. Radiation

The immunocytochemical staining of cultured NFs and KFs indicated that collagen 1/3 were downregulated by quercetin and X-rays. The mRNA levels of TGF-β1 and collagen 1/3 in both KFs and normal fibroblasts were reduced by quercetin, and cotreatment with X-rays further enhanced the effects of quercetin [87].

### 4.4. Pulsed-Dye Laser

A flash-lamp pulsed-dye laser treatment at a 585 nm wavelength at optimal dosages attenuated KF proliferation, TGF-β1 expression, and AP-1 activation [88]. The pulsed-dye laser treatment induced the phosphorylation of ERK1/2 and p38 MAPK in cells, and pre-treatment of cells with PD98059 (an ERK inhibitor) or SB203580 (a p38 MAPK inhibitor), but not SP600125 (a JNK inhibitor), abolished the effects of the pulsed-dye laser in downregulating c-Jun phosphorylation and TGF-β1 expression [88]. Thus, a pulsed-dye laser treatment could regress keloids by triggering cascades of certain subtypes of MAPK and a blockade of AP-1 activation and TGF-β1 expression.

### 4.5. Photodynamic Therapy (PDT)

PDT is used in treating hyperplastic dermatosis and keloids. PDT involving hematoporphyrin monomethyl ether (HMME) and light irradiation inhibited KF proliferation and increased the rate of apoptosis more strongly than irradiation alone, HMME treatment alone, or no treatment control [89]. The levels of ROS, detected using dihydroethidium (DHE) and dihydrorhodamine (DHR123), were higher in the PDT group than in the other three groups [89]. Thus, HMME-mediated PDT could affect KF proliferation and apoptosis by increasing ROS production.

## 5. Biological Activities of Plant Extracts in Cells

This section describes experimental observations from studies focusing on the biological activities of plant extracts in KFs. The proposed therapeutic targets and representative phytochemicals of plant-derived extracts are summarized in Table 1. The identity of the active compounds is known only for a few plant extracts, as shown in Table 1. For other plant extracts, general information on their phytochemicals is presented to help gain insight into their yet-to-be-identified active compounds.

The combined extract of *Astragalus membranaceus* and *Salvia miltiorrhiza* inhibited the proliferation, invasion, and collagen synthesis of KFs stimulated by TGF-β1 [90]. The extract inhibited the phosphorylation of SMAD2/3 on their linker regions (SMAD2L and SMAD3L) rather than the C-terminal regions (SMAD2C and SMAD3C). The extract blocked the formation and nuclear translocation of SMAD2/3/4 complexes, whereas it increased SMAD7 expression. As a result, the expression of a target gene, PAI-1, was suppressed at the transcription and translation levels.

From the screening of various natural products, the extract of *Aneilema keisak* (also called *Murdannia keisak*) was identified to effectively inhibit the nuclear translocation of SMAD 4 induced by TGF-β1 [94].The extract suppressed the TGF-β-mediated signaling pathway by inhibiting SMAD2 protein expression and phosphorylation. This extract attenuated key properties of KFs, such as cell proliferation, migration, and collagen synthesis, without causing DNA damage. It induced cell cycle arrest in the G_2_ phase and cellular senescence.

Wubeizi ointment (also called *Galla chinensis* ointment) contains several Chinese herbs, such as *Salvia miltiorrhiza*, *Galla Chinensis*, and *Lycium chinense* [96]. Wubeizi ointment inhibited proliferating KFs in a time- and dose-dependent manner [97]. It increased the proportion of cells in the S phase while reducing the proportion of cells in the G_2_/M phase. The ointment reduced the mRNA expression level of procollagen 1/3 in KFs [98]. Wubeizi ointment suppressed cell proliferation; induced apoptosis; decreased the mRNA, protein, and phosphorylation levels of AKT and mTOR; and increased PTEN mRNA and protein levels in KFs in the absence and presence of insulin-like growth factor 1 (IGF-1) in vitro [96]. The level of miR-21 was higher and the mRNA and protein levels of PTEN were lower in keloid tissue compared to normal tissue, and the Wubeizi ointment reduced miR-21 and increased PTEN mRNA and protein in keloid tissue [99].

The ethanol extract from *Physalis angulata* leaves lowered cell viability (IC_50_, 6.3 μg m L^−1^) [103]. The extract, at 0.63 μg mL^−1^, reduced the expression levels of collagen 1 from 47.866 ng mL^−1^ (control level) to 12.910 ng mL^−1^. The extract, at 2.51 μg mL^−1^, reduced the expression levels of tissue inhibitor of metalloproteinase 1 (TIMP-1) from 9.972 ng mL^−1^ (control level) to 5.350 ng mL^−1^. The expression levels of PAI-1 tended to decrease with the extract treatment but there was no statistical significance.

Onion extract (250 mg mL^−1^) inhibited the proliferation of the human fibroblast cell line (46 BR.1 N) (50.8% reduction) [105]. Green tea extract inhibited collagen 1 production by modulating the PI-3K/Akt/mTOR signaling pathway [107].

## 6. Biological Activities of Plant-Derived Compounds in Cells

**Figure 2:**
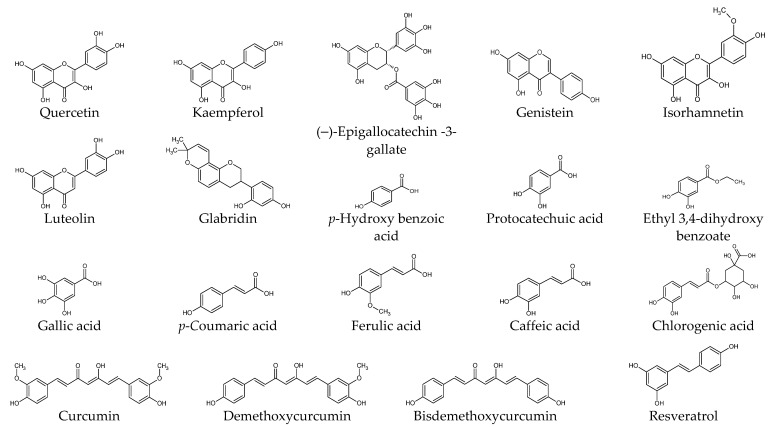

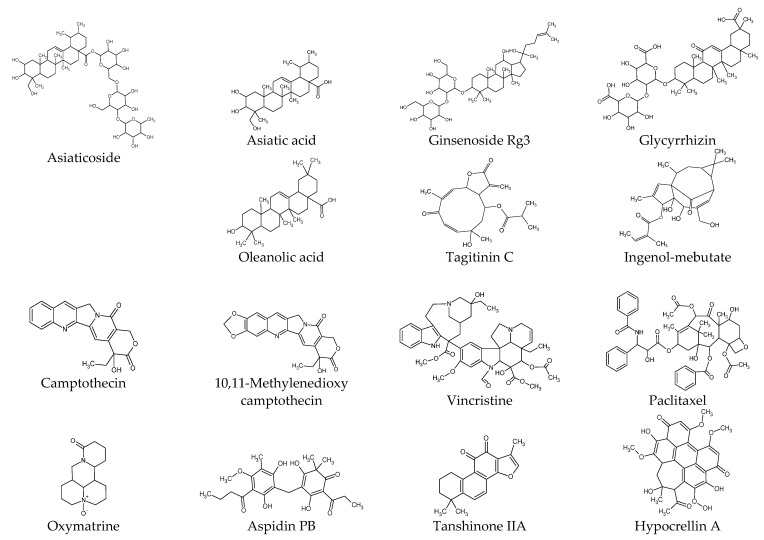
Chemical structures of natural products.

This section presents experimental results from studies on the biological activities of plant-derived compounds in KFs at the cellular and molecular level. The compounds are described by classifying them into phenolic compounds, terpenoids, alkaloids, and others. The chemical structure of each compound is shown in Figure 2. The proposed therapeutic targets of plant-derived compounds are summarized in Table 2.

### 6.1. Phenolic Compounds

Quercetin attenuated KF proliferation (IC_50_, 25 μg mL^−1^) and lowered the expression levels of TGF-β1, TGFβR1/2, collagen 1/3, and fibronectin [87,108,109,110]. It lowered the expression levels of SMAD2/3/4 and reduced the phosphorylation of SMAD2/3, and the formation of the SMAD2/3/4 complex [110]. It lowered the expression levels of the insulin-like growth factor 1 receptor (IGF-1R) β subunit, insulin receptor substrate (IRS) 1, PI3K p85 subunit, c-Raf, and reduced the phosphorylation of c-Raf, MEK1/2, ERK1/2, ETS like protein (ELK) 1, and AKT1 in KFs [108].

(–)-Epigallocatechin-3-gallate (EGCG) attenuated the proliferation, migration, and collagen production of KFs and NFs, and reduced the phosphorylation of STAT3, but not that of SMAD2/3, in KFs [59]. Green tea extract and EGCG lowered the expression level of collagen 1 and reduced the phosphorylation of AKT, eukaryotic translation initiation factor 4E-binding protein (4E-BP), and p70S6K in KFs stimulated by human leukemic mast cell line HMC-1 [107].

CTGF protein levels were higher in KFs compared to NFs, and genistein reduced the CTGF protein levels in KFs [112]. Genistein, at different concentrations (37 or 370 μM), had variable effects on the mRNA expression levels of subunit proteins of AP-1, such as c-Jun, c-Fos, and FosB, in skin keratinocytes, NFs, and KFs [131].

Luteolin decreased the KF viability and the expression levels of cyclin D1, BCL-2, and FRAT1, and increased cell apoptosis, p21, and BAX expression [113]. The pro-apoptotic effects of luteolin were abolished by overexpressed FRAT1, a GSKβ3 inhibitor causing β-catenin activation in the Wnt signaling pathway, and siRNA-mediated FRAT1 depletion increased cell apoptosis [113].

Glabridin, a component of *Glycyrrhiza glabra*, reduced KF proliferation and collagen production and induced apoptosis by inhibiting the PI3K/AKT and TGF-β1/SMAD signaling pathways in vitro [114].

Isorhamnetin inhibited the proliferation, migration, invasion, and fibrogenesis of KFs [74]. It lowered the expression level of S1PR1 and reduced the phosphorylation of PI3K and AKT [74]. S1PR1 upregulation abolished the inhibitory effects of isorhamnetin on KF proliferation, migration, invasion, and fibrogenesis.

KF proliferation was inhibited by curcumin (2.5 and 5 μg mL^−1^), gallic acid (5 and 10 μg mL^−1^), quercetin (10 and 20 μg mL^−1^), kaempferol (20 μg mL^−1^), protocatechuic acid (100 and 200 μg mL^−1^), p-coumaric acid (400 μg mL^−1^), ferulic acid (400 μg mL^−1^), and chlorogenic acid (400 μg mL^−1^) [111]. *p*-Hydroxy benzoic acid had no effect, and caffeic acid was very toxic [111]. These effects were attributed to cell cycle arrest rather than apoptosis [111]. The cell proliferation was resumed after the removal of each phytochemical and relatively slow recovery was seen with quercetin, chlorogenic acid, or curcumin [111]. Quercetin, gallic acid, protocatechuic acid, and chlorogenic acid more effectively inhibited the collagen lattice contraction by NFs and hypertrophic scar-derived fibroblasts (HSFs) than other compounds [111]. The collagen lattice contraction resumed when each compound was removed, and the recovery was slowest with quercetin [111].

Curcuminoids (25–100 nM), consisting of curcumin, demethoxycurcumin, and bisdemethoxycurcumin, lowered the cellular levels of total soluble collagens, pro-collagen 1, fibronectin, and TGF-β1, and reduced the phosphorylation of SMAD2 in KFs stimulated with bleomycin [115]. Curcumin was the major form of curcuminoids that entered and accumulated inside cells [115].

Resveratrol attenuated cell proliferation, induced apoptosis, and lowered the expression levels of TGF-β1, collagen 1, α-SMA, and heat shock protein (HSP) 47, which is involved in collagen folding and remodeling [36,132], in KFs but not in NFs [116]. It also attenuated cell proliferation, induced apoptosis, and reduced the collagen synthesis of KFs under hypoxia by downregulating hypoxia-inducible factor (HIF)-1α.

### 6.2. Terpenoids

Asiaticoside, a component of *Centella asiatica*, attenuated KF proliferation and lowered the expression levels of collagen 1/3 and TGFβR1/2 [117]. Asiaticoside did not affect the expression levels or the phosphorylation of SMAD2/3/4 but increased the expression level of SMAD7, which acts as an intracellular antagonist of the TGF-β signaling pathway [117]. Asiaticoside attenuated KF proliferation, invasion, and the phosphorylation of ERK1/2, p38 MAPK, and SMAD2/3 (linker region) stimulated by GDF-9 [82].

Asiatic acid from *Centella asiatica* suppressed the TGF-β1-induced expression of collagen 1 and plasminogen activator inhibitor-1 (PAI-1) and the phosphorylation of SMAD2/3, while increasing SMAD7 expression [118]. These effects of asiatic acid on KFs were abrogated by PPAR-γ antagonist GW9662 or peroxisome proliferator-activated receptor gamma (PPAR γ) siRNA [118].

Ginsenoside Rg3 (50 or 100 µg mL^−1^) attenuated the proliferation, migration, invasion, angiogenesis, and collagen synthesis of KFs and inhibited the TGF-β/SMAD and ERK-mediated signaling pathways [119].

Tagitinin C reduced KF viability after 72 h (IC_50_, 0.122 μg mL^−1^), as potently as mitomycin C (IC_50_, 0.120 μg mL^−1^) [120]. Tagitinin C at IC_50_ decreased keloid collagen deposition to 53.1% of the control level, whereas mitomycin C IC_50_ decreased it to 60.4% [120]. Tagitinin C and mitomycin C were less toxic to NFs (IC_50_; 35.05 μg mL^−1^ and 16.21 μg mL^−1^, respectively) [120]. The selective cytotoxicity index of tagitinin C and mitomycin C on KFs versus NFs was calculated to be 287 and 135, respectively [120].

Treatment of KFs with ingenol-mebutate induced morphological alterations and DNA fragmentation, which were associated with reduced cell growth and increased apoptosis [121]. It induced the expression of miR-34a in a p53-dependent manner and upregulated proapoptotic genes, such as caspase-10, while downregulating antiapoptotic genes, such as BCL-2 [121].

Glycyrrhizin, a component of *Glycyrrhiza glabra*, lowered the expression level of HMGB1 in KFs and attenuated cell proliferation and autophagy while increasing apoptosis [70]. Glycyrrhizin inhibited the expressions of ERK1/2, AKT, and NF-κB induced by HMGB1 [70]. Glycyrrhizin lowered the expression levels of TGF-β1, SMAD2/3, ERK1/2, collagen 1/3, fibronectin, and elastin in KFs [70].

Oleanolic acid attenuated the proliferation of KFs [122]. It lowered the expression levels of intra- and extracellular fibronectin, procollagen 1, and α-SMA while increasing MMP1 [122]. It inhibited the phosphorylation of SMAD2 and SMAD3 and attenuated the increases in fibronectin, procollagen 1, and α-SMA and the decrease in MMP1 in KFs stimulated with TGF-β1.

### 6.3. Alkaloids

Camptothecin, originally isolated from *Camptotheca acuminata*, is a topoisomerase inhibitor that has been used in cancer therapy [133]. Camptothecin lowered the expression levels of collagen 1/3 in KFs without causing cellular toxicity [123]. Its effects on the collagen 3 level were relatively smaller, and consequently, the ratios of collagen 1 to collagen 3 were decreased by the camptothecin treatment [133].

10,11-Methylenedioxycamptothecin loaded in hyaluronic acid nanoemulsions were delivered percutaneously to the keloid lesion area in a mouse model [124]. Its internalization by KFs and delivery to the nucleus resulted in decreased cell proliferation [124]. It increased the expression levels of TGF-β1, SMAD3, and SMAD7, and downregulated PAI-1 in KFs, implicating an overall suppression of the TGF-β-mediated signaling pathway [124].

Oxymatrine, an alkaloid compound extracted from *Sophora japonica*, lowered the expression levels of collagen and SMAD3 in KFs in vitro without affecting the expression levels of TGF-β1, TGFβR1/2, SMAD4, and SMAD7 [125]. Oxymatrine inhibited the phosphorylation and nuclear translocation of SMAD3 induced by TGF-β1 [125]. Thus oxymatrine could attenuate collagen synthesis by inhibiting the TGF-β/SMAD signaling pathway.

Vincristine is one of the vinca alkaloids originally separated from *Catharanthus roseus*, and is used as an anticancer drug [134]. Vincristine inhibited cell proliferation by inducing cell cycle arrest in the G2/M phase and promoting apoptosis in SH-SY5Y human neuroblastoma cells [135]. Vincristine showed cytotoxicity to the primary KFs and NFs, with higher potency to the latter [24]. The resistance of KFs could be largely abrogated by verapamil (a calcium channel blocker) [24].

The treatment of KFs with paclitaxel or LY294002 (a PI3K inhibitor) lowered their expression levels of TNF-α, IL-6, TGF-β1, α-SMA, and collagen 1 [126]. Paclitaxel also blocked the AKT/GSK3β signaling pathway in KFs and keloid tissues [126]. Paclitaxel-cholesterol-loaded liposomes inhibited KF proliferation, migration, and invasion, and promoted apoptosis and cell cycle arrest in the G_2_/M phases more effectively than paclitaxel itself in vitro [126]. Paclitaxel-cholesterol-loaded liposomes had better performance in inhibiting keloid growth compared to paclitaxel in the keloid-bearing BALB/c nude mouse model [126].

### 6.4. Other Compounds

Aspidin PB inhibited the expression of collagen 1, CTGF, and α-SMA in KFs stimulated by TGF-β1 [127]. It inhibited both the SMAD2/3-mediated signaling pathway and the PI3K/AKT-mediated signaling pathway stimulated by TGF-β1 [127].

Tanshinone IIA attenuated the proliferation of KFs, whereas it did not affect the proliferation of NFs [128]. It increased the percentages of KF cells in the G_0_/G_1_ phases and the cells undergoing early apoptosis [128]. It also decreased the expression of survivin [128].

A selenium-containing polysaccharide from Ziyang green tea (Se-ZGTP) or short hairpin RNA (shRNA) for neuron-glia 2 inhibited the proliferation of KFs [129]. Se-ZGTP or NG2 shRNA induced apoptosis mediated by an increase in pro-apoptotic BAX expression, the activation of caspase-3, the subsequent cleavage and inactivation of poly (ADP-ribose) polymerase (PARP), and a decrease in the expression levels of anti-apoptotic BCL-2 [129]. Se-ZGTP or neuron-glia 2 shRNA reduced collagen 1 and protein expression in KFs following TGF-β1 stimulation [129].

As a photodynamic therapy, the combined treatment of hypocrellin A with a light-emitting diode (LED)’s red light irradiation increased ROS production [136] and decreased KF viability, proliferation, invasion, collagen production, and the expression of collagen 1/3, α-SMA, and fibronectin, while increasing cell apoptosis and the expression of BAX and caspase-3 [130]. The combined treatment reduced autophagy, the protein expression of Beclin-1, and the conversion of LC3-I to LC3-II [130]. It inhibited the expression of TGF-β and the downstream signaling pathways mediated by ERK1/2 and SMD2/3 [130].

## 7. Ex Vivo and In Vivo Studies

This section presents experimental results from ex vivo and in vivo studies evaluating the biological effects of plant-derived natural products on keloids.

### 7.1. Ex Vivo Studies

EGCG reduced cell proliferation, the expression levels of collagen 1/3, and keloid volume, but increased apoptotic cells in ex vivo experiments using an organ culture of keloid explants on a collagen matrix at an air–liquid interface [85]. It induced epidermal shrinkage and reduced the cellularity, blood vessel count, and keloid-associated mast cells.

Keloid tissue explants from patients were cultured ex vivo in this medium with, or without, ginsenoside Rg3 at varied concentrations. Histological examination indicated that ginsenoside Rg3 reduced angiogenesis and collagen accumulation in keloids [119].

### 7.2. In Vivo Studies

NFs and KFs were implanted into the backs of nude mice and EGCG was administered via intralesional injection [59]. At day 17, the size of the nodules derived from KFs was two times bigger than those derived from NFs. The results showed that EGCG markedly suppressed the increases in the size and collagen production of keloidal nodules in vivo.

The anti-scarring effect of glabridin was evaluated in a rabbit ear hyperplastic scar model, and histological examination indicated that glabridin reduced hyperplasia, inflammation, and collagen production in vivo [114].

Wubeizi ointment suppressed keloid growth in an in vivo model generated by implanting human keloid scar fragments into the back of nude mice [96]. Wubeizi ointment decreased the mRNA, protein, and phosphorylation levels of AKT and mTOR while increasing PTEN mRNA and protein levels in the keloid tissues of mice [96].

Electrospun ultrafine polymer fiber meshes loaded with dexamethasone and green tea polyphenols effectively induced the degradation of collagen fibers and keloid volume shrinkage, compared to a traditional treatment (silicone gel sheeting), in animal keloid models established by subcutaneously implanting human keloid tissues into the backs of athymic nude mice [137].

## 8. Clinical Studies

This section presents observations from clinical trials of several natural products.

### 8.1. Silicone Gel versus Tretinoin Cream

The efficacies of silicone gel and tretinoin cream in the treatment of hypertrophic scars and keloids were compared in a study of 26 patients with 44 different wounds [138]. The patients applied silicone gel, tretinoin cream, or neither twice a day on the wounds for up to 24 weeks after removal of stitches. The silicone gel and tretinoin cream were comparably effective in reducing hypertrophic scars and keloids compared with the control group, and there were no significant differences between the groups treated with the silicone gel or tretinoin cream.

### 8.2. Silicone Gel versus Onion Extract, or Their Combination

The therapeutic activities of topical onion extract, a silicone gel sheet, and a combination of onion extract and a silicone gel sheet were compared in a prospective clinical study, and assigned to 60 patients [139]. The onion extract improved the scar color more effectively, while the silicone gel sheet reduced the height of the scar more effectively. A combination of the onion extract and silicone gel sheet was the most effective in improving hypertrophic and keloid scars.

### 8.3. Onion Extract Gel versus Placebo in Adult Patients

In a randomized, double-blinded, placebo-controlled study involving 60 patients who underwent median sternotomy, a gel composed of a silicone derivative and onion extract (major active component, quercetin) (Cybele scagel) was demonstrated to be safe and effective for preventing hypertrophic scarring, as it reduced the pain and itch score and pigmentation compared to the placebo gel, although there were no differences in the scores for vascularity, pliability, and height between these treatments [106].

### 8.4. Onion Extract Gel versus Placebo in Pediatric Patients

The efficacy of a silicone-derivative gel containing 10% onion extract was compared with a placebo gel in a prospective, randomized, double-blind, placebo-controlled split-scar trial study in 39 pediatric patients who underwent median sternotomy [140]. Six patients (20%) in the onion extract gel group showed no scars, and one patient (3.3%) in the placebo group showed no scars. Of the 27 patients with hypertrophic scars, 9 were in the onion extract gel group and 18 were in the placebo group. There was no statistically significant difference in keloids and on the Vancouver scar scale (VSS) between the two groups.

## 9. Discussion

### 9.1. Therapeutic Targets of Plant-Derived Extracts and Compounds

In this review, I presented studies on the biological activities and mechanisms of action of various natural products and plant extracts which can potentially inhibit the development of keloids. It has been suggested that each substance prevents the excessive proliferation of cells by causing cell cycle arrest or apoptosis, and blocks the accumulation of ECM components, such as collagen, by inhibiting various cell signaling pathways.

**Figure 3:**
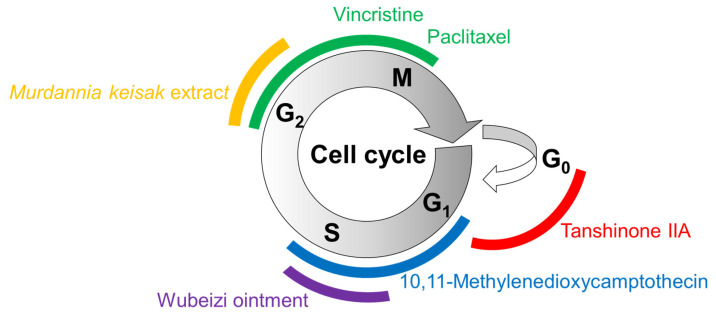
Cell cycle arrest by plant-derived extracts and compounds. The cell cycle consists of gap (G_1_) phase, synthesis (S) phase, (G_2_) phase, mitosis (M) phase, and (G_0_) phase. Different colors are used to represent different phases of cell cycle arrest induced by the treatments with matching colors.

**Figure 4:**
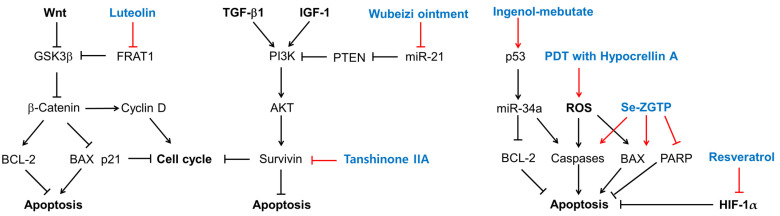
Potential mechanisms for cell cycle arrest and apoptosis induction of several plant-derived extracts and compounds in KFs. Sharp arrows indicate stimulation or upregulation and blunted arrows indicate inhibition or downregulation. Black lines indicate the interaction between cellular factors and events (black letters) and red lines indicate the effects of various treatments (blue letters).

The effects of several plant-derived extracts and compounds on the cell cycle are shown in Figure 3. Their potential mechanisms of action for inducing cell cycle arrest or apoptosis are summarized in Figure 4.

Luteolin inhibits the Wnt signaling pathway by promoting the degradation of β-catenin by downregulating FRAT1, which inhibits the GSK3β-mediated phosphorylation of β-catenin [113]. As a result, luteolin induces cell cycle arrest or apoptosis, which is associated with low levels of cyclin D1 and BCL-2 and high levels of p21 and BAX [113]. Wubeizi ointment induces cell cycle arrest by increasing phosphatase and tensin homolog (PTEN) through the downregulation of miR-21 [96,97,98,99], which results in the inhibition of the AKT/mTOR pathway. Tanshinone IIA induces cell cycle arrest or apoptosis by lowering survivin, which is an essential protein for cell division and an inhibitor of cell death [141]. Ingenol-mebutate upregulates a range of miRNAs including p53, miR-34a, BCL-2, and caspases, which can induce cell cycle arrest or apoptosis [142]. PDT with hypocrellin A increases ROS production and apoptosis by upregulating BAX and caspase-3. Se-ZGTP induces apoptosis, which is accompanied by a decrease in BCL-2 and increases in BAX and caspase-3, and the subsequent cleavage and inactivation of PARP that mediates the repair of DNA damage [143]. Resveratrol induces apoptosis by downregulating HIF-1α, with antiapoptotic effects.

**Figure 5:**
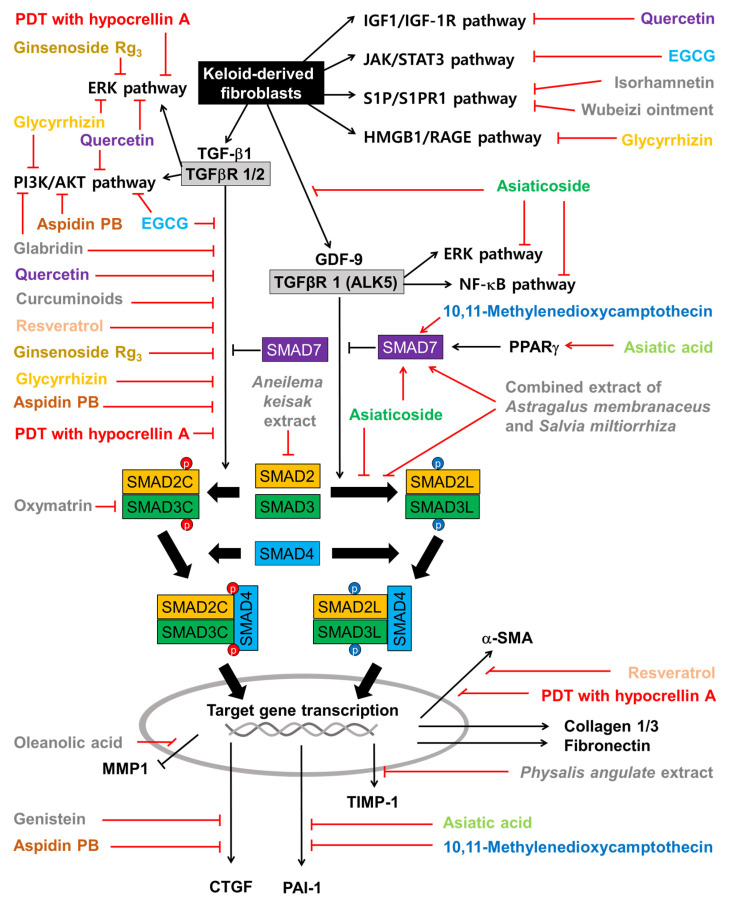
Effects of plant-derived extracts and compounds on cell signaling pathways in KFs. Sharp arrows indicate stimulation or upregulation and blunted arrows indicate inhibition or downregulation. Black lines indicate the interaction between cellular factors and events (black and white letters) and red lines indicate the effects of various plant-derived extracts and compounds (different color or grey letters).

The effects of plant-derived extracts and compounds on the cell signaling pathways in KFs are summarized in Figure 5. Among the flavonoids, quercetin inhibits not only the cell signaling pathways induced by TGF-β1 but also those induced by IGF-1. Isorhamnetin inhibits the S1P/PI3K/AKT signaling pathway. EGCG inhibits both the TGF-β signaling pathways and the JAK/STAT pathway. Glabridin inhibits both the TGF-β1/SMAD pathway and the PI3K/AKT pathway. Genistein inhibits the expression of CTGF, which is a target gene of the TGF-β1/SMAD pathway. Among other phenolic compounds, curcuminoids and resveratrol inhibit the TGF-β1/SMAD pathway.

Among the terpenoids, asiatic acid and asiaticoside inhibit the TGF-β1/SMAD pathway by increasing SMAD7 in a PPARγ-dependent mechanism. Asiaticoside inhibits the phosphorylation of SMAD2/3 (linker regions) stimulated by GDF-9. Asiatic acid downregulates PAI-1, which is a target gene of the TGF-β1/SMAD pathway and an inhibitor of the proteolytic activation of plasminogen to plasmin [144]. Ginsenoside Rg3 inhibits the TGF-β/SMAD and ERK-mediated signaling pathways. Glycyrrhizin inhibits the activation of the ERK1/2, AKT, and NF-κB pathways in response to the HMGB1 signal transmitted through RAGE (or TRLs). Oleanolic acid restores MMP1 downregulated by TGF-β1. Among the alkaloids, 10,11-methylenedioxycamptothecin inhibits the TGF-β1/SMAD pathway and PAI-1 expression by increasing SMAD7. Oxymatrine attenuates the TGF-β1/SMAD pathway by lowering SMAD3 expression, phosphorylation, and nuclear translocation. And aspidin PB inhibits both canonical and non-canonical TGF-β signaling pathways and the expression of CTGF.

The combined extract of *Astragalus membranaceus* and *Salvia miltiorrhiza* inhibits the phosphorylation of SMAD2/3 on the linker regions and increases SMAD7 levels, suppressing PAI-1 expression. The extract of *Aneilema keisak* inhibits the TGF-β-mediated signaling pathway by inhibiting SMAD2 expression. The extract of *Physalis angulata* inhibits the expression of TIMP-1.

### 9.2. The Potential of Natural Product-Based Therapy and Future Tasks

Recognizing reports of side effects such as increased keloid formation during the implementation of existing treatments [145,146], natural-product-based treatments have the potential to be safe treatments that can replace or supplement existing treatments. However, it is believed that research on natural-product-based treatments is far from reaching a level of maturity that allows for their clinical application.

Many of the studies presented in this review were performed using patient-derived KFs and NFs, which reflects the phenotypic differences between the two cells. KFs showed higher responsiveness to TGF-β1 stimulation than NFs in terms of proliferation, migration, invasion, and collagen synthesis [118]. KFs were more resistant to apoptosis than NFs and neutralizing antibodies to TGF-β1 abrogated their difference in apoptosis resistance [23]. Blocking IGF-1 or IGF-1R with their respective specific antibodies inhibited KF proliferation [108]. Thus, an experimental approach using KFs is believed to be appropriate and has greatly contributed to our understanding of the mechanism of action of the test substances.

However, only a few studies have progressed to the in vivo experiment stage, which is essential to verify their efficacy. This may be because there is no available animal model that shows the spontaneous development of keloids after injury. In ex vivo experiments in which human keloid explants were artificially cultured, only EGCG and ginsenoside Rg3 were tested. Additionally, the efficacy of EGCG and Wubeizi ointment was evaluated in an in vivo model in which a piece of human keloid tissue, or cells isolated from it, was subcutaneously injected into a mouse or a full-thickness skin graft was transplanted into the incised skin of a mouse. The efficacy of ultrafine fiber meshes loaded with dexamethasone and green tea polyphenols was evaluated in animal models and compared with conventional silicone gel. The stabilization or improvement of existing in vivo animal models and the development of new animal models are expected to enable more physiologically relevant research.

There are very few clinical studies available. Silicone gel and tretinoin cream showed similar efficacy in relieving hypertrophic scars and keloids in clinical trials, and silicone gel containing onion extract, with quercetin as an active ingredient, was also demonstrated to have similar efficacy. Onion extract gel was particularly effective in improving skin color and alleviating hypertrophic scarring, but its efficacy in treating keloids could not be concluded as significant. We look forward to evaluating ultrafine fiber meshes loaded with dexamethasone and green tea polyphenols in clinical trials. When these ultrafine fiber meshes are used as surgical dressings, they will provide additional advantages through maintaining a moist environment, preventing bacterial infection, and controlling drug release [137].

It is necessary to utilize coculture experiments of different cells to overcome the limitations of single-cell models. The interaction between keratinocytes and fibroblasts was shown to be important for the regulation of cell proliferation and gene expression [147,148]. When keratinocytes or fibroblasts derived from either normal skin tissue or keloid tissues (Termed NKs, KKs, NFs, KFs, respectively) were cultured using transwell culture dishes, the combined culture of KKs and KFs more effectively increased the proliferation of fibroblasts than any other combination, and this effect was attenuated by a pan-TGF-β neutralizing antibody [147]. The coculture of KKs and KFs increased the gene expression of TGF-β1, TGF-β3, and TGFβR1 in keratinocytes, and TGF-β1, TGF-β2, TGFβR1, SMAD2, collagen 1, CTGF, and IGF-2/mannose-6-phosphate receptor in fibroblasts. These data suggest that keloid pathogenesis may involve communication between KKs and KFs.

NKs, KKs, NFs, and KFs express different levels of activins and follistatin (an inhibitor of activins), and their interaction can affect the pathogenesis of keloids [76]. NFs cocultured with HaCaT keratinocytes showed a higher proliferation than non-cocultured cells, and the proliferation was further enhanced when NFs were cocultured with HaCaT cells overexpressing activin-A [76].

Keloid development involves a local infiltration of inflammatory cells including mast cells [7]. Cocultured HMC-1 stimulated the collagen 1 expression of KFs, and it was attenuated by a blockade of PI3K, mTOR, and p38 MAPK [107]. Thus, the interaction between mast cells and fibroblasts may contribute to excessive collagen production in keloids. This coculture model has been used in evaluating the biological activity of EGCG [107]. The coculture model may provide a more physiologically relevant model than a monocellular culture model in studying keloid pathogenesis and intervention strategies.

### 9.3. Emerging Therapeutic Targets and Future Perspectives

Except for a study comparing the inhibitory cell proliferation effects of various phenolic compounds [111] and a study comparing the effects of various plant extracts that inhibit the SMAD signaling system [94], most studies were limited to tests of one or two substances, making it difficult to identify better drug candidates that are superior to others. Future research should expand to the level of comparing the biological activities of various substances targeting a certain specific target to find the best drug candidates. In the case of plant extracts, it is necessary to identify the active ingredients in follow-up experiments and to compare the efficacy of these ingredients with existing drugs or to evaluate their efficacy by combining them with other therapies.

In addition to the well-established therapeutic targets, such as cell proliferation, apoptosis, the TGF-β signaling pathway, collagen synthesis pathway, and NF-κB signaling pathway, the newly proposed targets, such as the Wnt signaling pathway, JAK/STAT signaling pathway, HIF-1α, PGE_2_, AKR1B10, HMGB1, the S1P signaling pathway, activins and inhibins, and the GDF-9 signaling pathway, appear to be useful in research and drug discovery for keloid diseases. Instead of targeting the TGF-β signaling pathway directly, targeting its downstream target genes such as CTGF, PAI-1, and TIMP-1 would be a good alternative for keloid treatment.

In particular, AKR1B10, revealed to be a differentially overexpressed gene in keloids through the whole-genome microarray analysis of lesions and normal tissue, and GDF-9, discovered through analysis using a TGF-β superfamily signaling gene chip, are attractive therapeutic targets [67,80]. In addition, microRNAs, such as miR-21, which shows differential overexpression in keloids, and miR-34a, which shows changes in expression levels in response to drugs, can also be potential therapeutic targets to control epigenetic changes in diseases [99,121].

Keloid cells exhibit the phenotypic characteristics of rapid proliferation, sustained autophagy, and suppressed apoptosis. Therefore, in addition to suppressing proliferation or promoting apoptosis, targeting autophagy can also be a useful strategy for keloid treatment. Autophagy is a cellular clearance and recycling process, essential for cell survival and homeostasis under normal, nutrient-deficient, or stressful conditions [149,150].

Immunohistochemical analysis of keloid tissues indicated that vimentin-expressing fibroblasts in the central zone undergo autophagy and glycolysis at higher levels than those in the marginal zone, which was associated with the expression level of HIF-1α [151]. These observations suggest that targeting the autophagy and glycolysis of fibroblasts in hypoxic-zone keloids may have therapeutic implications in keloid treatment. Autophagy inhibitor 3-methyladenine decreased the collagen levels in NFs stimulated with TGF-β1 [70]. It is noteworthy that glycyrrhizin treatment or PDT with hypocrelin A could suppress the autophagy of KFs [70,130].

### 9.4. Subjective Opinions

Although it is difficult to know which of the keloid therapeutic targets is the most promising, we recommend paying attention to GDF-9, which controls cell proliferation, in addition to TGF-β1, which controls ECM production. There is no need to be limited to one therapeutic target, and multi-targeting should also be considered. Strategic judgment is required to decide whether to develop individual compounds or plant extracts, as each has pros and cons. In the case of edible plant extracts, clinical application can be quick and easy, and, since they are a mixture of various compounds, multifunctionality can be expected. Complementing and improving the currently used herbal treatments, such as Wubeizi ointment, onion extract, and green tea extract, may also be an option. In addition, I believe that the harmonious development of the epidermis and dermis in wound healing may minimize the occurrence of scars. To this end, research will be needed to understand the cross-talks between epidermal and dermal cells and to develop strategies to fine-tune their relative proliferation and development. A customized treatment strategy tailored to the patient’s post-injury keloid progression stage is also necessary for satisfactory outcomes.

## 10. Conclusions

Keloids are benign skin tumors occurring as a result of the deregulated wound healing process and are characterized by high cell proliferation, high autophagy, low apoptosis, high angiogenesis, high production of collagen, and activation of the TGF-β cell signaling pathways. Many plant-derived extracts or compounds attenuate cell proliferation by inducing cell cycle arrest or increasing apoptosis. Some of them also attenuate collagen synthesis. They can intervene in various signaling pathways stimulated by TGF-β1, IGF-1, S1P, GDF-9, HMGB1, Wnt, or cytokines, and mediated by SMADs, ERK1/2, p38 MAPK, JNK, PI3K/AKT, JAK/STAT, NF-κB, or β-catenin. They downregulate FRAT1, cyclin D, miR-21, survivin, BCL-2, or PARP, and upregulate PTEN, p53, miR-34a, BAX, or caspases to induce cell cycle arrest or apoptosis. They downregulate fibrogenic factors, such as CTGF, HSP47, TIMP-1, and PAI-1, and upregulate proteolytic factors, such as MMP1. For now, many studies are limited to in vitro experiments; additional research and development are needed to proceed to clinical trials. The lack of ideal animal models in which keloids develop naturally after skin injury remains an obstacle to research in this field. On the other hand, many emerging therapeutic targets could accelerate the discovery of plant-derived bioactive compounds for the prevention and treatment of keloid disease.

## Figures and Tables

**Table 1:** The therapeutic targets and phytochemicals of plant-derived extracts.

Extracts	Therapeutic Targets	Plants	Phytochemical Information
Combined extract of Astragalus membranaceus and Salvia miltiorrhiza	Proliferation, Invasion, Collagen synthesis, SMAD pathway, PAI-1 [90]	*Astragalus membranaceus*	Contains flavonoids, such as quercetin, kaempferol, genisteine, dihydroxyflavone, and liquiricigenin, and terpenoids, such as astragaloside I, astragaloside II, astragaloside IV, isoastragaloside I, and isoastragalosides II [91,92].
		*Salvia miltiorrhiza*	Contains phenolic compounds, such as danshensu, salvianolic acid A, salvianolic acid B, protocatechuic aldehyde, rosmarinic acid, and caffeic acid, and terpenoids, such as tanshinone IIA, cryptotanshinone, dihydrotanshinone, isotanshinone I, isotanshinone IIA, and isocryptotanshinone [93].
Aneilema keisak extract	Proliferation, Migration, Collagen synthesis, SMAD pathway, Cell cycle arrest, Senescence [94]	*Aneilema keisak*	Contains phenolic compounds, such as latifolicinin A, latifolicinin B, latifolicinin C, protocatechuic acid, hydroxytyrosol, oresbiusin A, kaempferol, epigallocatechin, rutin, ferulic acid, vanillic acid, chlorogenic acid, and p-coumaric acid [95].
Wubeizi ointment	Proliferation, Cell cycle arrest, Apoptosis, AKT pathway, IGF-1, miR-21, PTEN [96,97,98,99]	*Salvia miltiorrhiza*	See above.
		*Galla Chinensis*	Contains a variety of tannin compounds, such as 1,2,6-tri-O-galloyl-β-D-glucose and 1,2,3, 6-tetra-O-galloyl-β-D-glucose, and other phenolic compounds, such as methyl gallate, ethyl gallate, ellagic acid, myricetin-3-O-rhamnoside, and epigallocatechin [100].
		*Lycium chinense*	Contains rutin and chlorogenic acid [101]. It also contains various polysaccharides, carotenoids, alkaloids, and phenolic compounds [102].
Physalis angulate extract	Cell viability, Collagen synthesis, TIMP-1 [103]	*Physalis angulate*	Contains phytosterols, such physalins A–I, physagulin A–G, withangulatin A, and withanolide T [104].
Onion exract	Cell proliferation [105]	*Allium cepa*	Contains quercetin as the main active compound [105,106].
Green tea extract	Collagen synthesis, AKT pathway [107]	*Camellia sinensis*	Contains (–)-epigallocatechin-3-gallate (EGCG) as the main active compound [107].

**Table 2:** Proposed therapeutic targets of plant-derived extracts and compounds. The targets modulated by each compound are indicated by check mark (√).

Compounds	Proliferation/ Viability	Migration/ Invasion	Apoptosis	ECM Production	TGF-β Level	TGFβR Level	SMAD Pathway	AKT Pathway	ERK Pathway	Additional Targets	Literature
Quercetin	√			√	√	√	√	√	√	IGF1R	[87,108,109,110,111]
Kaempferol	√										[111]
(–)-Epigallocatechin -3-gallate	√	√		√				√		STAT3	[59,85,107]
Genistein										CTGF	[112]
Luteolin	√		√							FRAT1	[113]
Glabridin	√		√	√			√	√			[114]
Isorhamnetin	√	√		√				√		S1PR1	[74]
Protocatechuic acid	√			√							[111]
Gallic acid	√			√							[111]
p-Coumaric acid	√										[111]
Ferulic acid	√										[111]
Chlorogenic acid	√			√							[111]
Curcumin	√			√	√		√				[111,115]
Demethoxycurcumin				√	√		√				[115]
Bisdemethoxycurcumin				√	√		√				[115]
Resveratrol	√		√	√	√		√			HSP47, α-SMA	[116]
Asiaticoside	√	√		√		√	√		√	p38, GDF-9	[117]
Asiatic acid				√			√			PAI-1, PPARγ	[82,118]
Ginsenoside Rg3	√	√		√			√		√	Angiogenesis	[119]
Tagitinin C	√			√							[120]
Ingenol-mebutate	√		√							miR-34a	[121]
Glycyrrhizin	√		√	√	√		√	√	√	NF-κB, HMGB1, Autophagy	[70]
Oleanolic acid	√			√			√			MMP1	[122]
Camptothecin				√							[123]
10,11-Methylenedioxy camptothecin	√				√		√			PAI-1	[124]
Oxymatrine				√			√				[125]
Vincristine			√								[24]
Paclitaxel	√		√	√	√			√			[126]
Aspidin PB				√			√	√		CTGF	[127]
Tanshinone IIA	√		√							Survivin	[128]
Selenium- polysaccharide	√		√							PARP	[129]
Photodynamic therapy with Hypocrellin A	√	√	√	√	√		√		√	Autophagy, α-SMA	[130]

## Data Availability

Not applicable.

## Abbreviations

AKR1B10	Aldo-keto reductase family 1 member B10
AKT	protein kinase B
AP-1	activator protein-1
COX	cyclooxygenase
CTGF	connective tissue growth factor
DKK1	dickkopf-1
ECM	extracellular matrix
EGCG	(–)-epigallocatechin-3-gallate
ERK	extracellular signal-regulated kinase
FAS	Fas receptor
FRAT1	frequently rearranged in advanced T-cell lymphomas 1
GDF-9	growth differentiation factor-9
GSK3β	glycogen synthase kinase 3 beta
HIF	hypoxia-inducible factor
HMGB1	high-mobility group box 1
HMME	hematoporphyrin monomethyl ether
HMOX	heme oxygenase
HSP	heat shock protein 1
IGF-1	insulin-like growth factor 1
IGF-1R	insulin-like growth factor 1 receptor
IKK	IκB kinase
IL-6	Interleukin-6
INHBA	inhibin beta A
IκBα	NF-κB inhibitor
JAK	Janus kinase
JNK	c-Jun N-terminal kinase
KFs	keloid-derived fibroblast
KKs	keloid-derived keratinocyte
MAPK	mitogen-activated protein kinase
MEK	mitogen-activated protein kinase kinase
miRNA	micro RNA
MMP	matrix metalloproteinase
mTOR	mammalian target of rapamycin
NF-κB	nuclear factor kappa B
NFs	normal skin-derived fibroblasts
NKs	normal skin-derived keratinocytes
p70S6K	ribosomal protein S6 kinase
PAI-1	plasminogen activator inhibitor-1
PARP	poly (ADP-ribose) polymerase
PG	prostaglandin
PI3K	phosphoinositide 3-kinase
PKB	protein kinase B
PPARγ	peroxisome proliferator–activated receptor gamma
PTEN	phosphatase and tensin homolog
RAGE	receptors for advanced glycation end-products
ROS	reactive oxygen species
S1P	sphingosine-1-phosphate
S1PR	sphingosine-1-phosphate receptor
Se-ZGTP	selenium-containing polysaccharide from Ziyang green tea
SFRP	secreted frizzled-related protein
shRNA	hairpin RNA
siRNA	silencing RNA
α-SMA	alpha-smooth muscle actin
SMAD	small mothers against decapentaplegic
STAT	signal transducer and activator of transcription
TGF-β	transforming growth factor beta
TGFβR	TGF beta receptor
TIMP-1	tissue inhibitor of metalloproteinase 1
TLR	Toll-like receptor
TNF-α	tumor necrosis factor-alpha
